# Knowledge, Attitude, and Food Safety Practices among Street Food Vendors at a Metropolitan District in Ghana: A Cross-sectional Study

**DOI:** 10.1155/2024/5553942

**Published:** 2024-03-08

**Authors:** Abraham Norman Nortey, Hubert Amu, Ebenezer Senu, Alfred Effah

**Affiliations:** ^1^Department of Development Policy, Ghana Institute of Management and Public Administration, Accra, Ghana; ^2^Department of Population and Behavioral Sciences, Fred N. Binka School of Public Health, University of Health and Allied Sciences, Hohoe, Ghana; ^3^Department of Molecular Medicine, School of Medicine and Dentistry, Kwame Nkrumah University of Science and Technology, Kumasi, Ghana

## Abstract

**Background:**

Street food is a vital component of cities and towns in developing countries. However, food poisoning has been associated with inadequate knowledge of food safety practices and inappropriate food handling. We examined the knowledge, attitude, and practices of street food sellers in the Takoradi Submetropolis, Ghana, on food safety and hygienic practice.

**Method:**

In this descriptive cross-sectional study, 406 street food vendors were recruited based on a simple random sampling technique from the Sekondi-Takoradi Metropolis, Ghana, using a structured questionnaire. Data collected were analyzed with the chi-square test and binary logistic regression using Stata (version 16) software. Statistical significance was set at *p* < 0.05.

**Results:**

The level of knowledge was low among 70.4% of the food vendors, and 51% had negative attitudes towards food safety and hygiene. Food hygiene practices were also poor among 52.3% of the participants. The predictors of low knowledge level were senior high (aOR = 0.37, 95% CI (0.19-0.70), *p* = 0.002) and junior high education (aOR = 0.52, 95% CI (0.27-0.99), *p* = 0.047). Having senior high education (aOR = 0.37, 95% CI (0.17-0.82), *p* = 0.014), prior training on food safety and hygiene (aOR = 0.50, 95% CI (0.29-0.84), *p* = 0.010), and having high level of knowledge (aOR = 0.33, 95% CI (0.20-0.54), *p* = 0.001) were associated with lower likelihood of negative attitude towards food safety and hygiene. Moreover, having junior high education (aOR = 6.20, 95% CI (2.78-13.87), *p* = 0.001), high level of knowledge (aOR = 4.70, 95% CI (2.77-7.98), *p* = 0.001), and positive attitude towards food safety and hygiene (aOR = 1.76, 95% CI (1.08-2.87), *p* = 0.023) were associated higher odds of good food practice.

**Conclusion:**

Knowledge and attitude regarding food safety and hygienic practices was poor among street food vendors. Future initiatives should focus on establishing training programs for food vendors within the metropolitan assembly to improve their knowledge on food safety and hygienic practices.

## 1. Introduction

Food safety and hygiene are global health concerns, particularly in low- and middle-income countries (LMICs), due to the increasing prevalence of foodborne infections and associated mortality [1]. Food safety is a major concern, with outbreaks of foodborne illness costing individuals, the food industry, and the economy significantly [2]. Despite efforts by governments in these LMICs to promote food safety, the prevalence of foodborne disease remains a significant health hazard in both industrialized and developing countries [2]. According to the WHO, 600 million foodborne diseases (FBDs) occur each year as a result of poor food safety and hygiene practices [3]. This results in 420,000 deaths, the majority of which are due to meat-related vulnerabilities [3, 4]. In the United States, approximately 76 million FBDs resulted in 325,000 hospitalizations and 5000 deaths [5]. In Ghana, FBDs kill approximately 65,000 people each year, including 5000 children under the age of five [1, 6]. According to a recent survey, a total of 1914 Ghanaians experienced various types of food poisoning between 2013 and 2021 [7]. Moreover, about 60 cases from 36 facilities resulted in 36 patients losing their lives [7]. Enterobacter, Citrobacter, Klebsiella, Shigella, and Escherichia coli have been identified as the most prevalent foodborne pathogens in Ghanaian street-vended food [8]. Previous studies have also reported mesophilic bacteria and Enterobacteriaceae above acceptable levels in ready-to-eat street-vended foods in Ghana [9, 10]. Given that the majority of LMIC inhabitants depend mainly on street foods, the role of the street food sector cannot be undermined.

The street food sector plays a critical role in satisfying the dietary needs of commuters and urban dwellers in many developing-country cities and towns, feeding thousands of people every day with a diverse choice of dishes that are relatively inexpensive and easily accessible [6, 11]. However, many of these street foods do not satisfy basic hygienic standards, which can result in morbidity and mortality from foodborne illnesses, as well as associated repercussions on trade and development. The main causes of contamination of ready-to-eat foods are the use of conventional processing techniques such as grinding and milling, improper holding temperatures, and inadequate personal hygiene on the part of food handlers [11]. Moreover, the food is not sufficiently shielded from dust and flies [11]. Street food in Ghana is primarily made and processed by hand and sold to the public at various lorry terminals, by the roadway, or by itinerant sellers. Most of these roadways and truck terminals are usually choked with dirt, which predisposes the food to flies.

Ghana is a developing nation renowned for its delicious, appealing, and varied street cuisine. In addition to satisfying the appetites of locals like those in the Takoradi Submetropolis of the Sekondi-Takoradi Metropolitan Assembly (STMA), Ghana's abundance of delicacies also piques the interest of tourists. Although there have been a considerable number of episodes of food poisoning linked to street meals in recent years [12], there is paucity of data regarding food safety and hygiene practices among street food vendors with STMA. Therefore, this study assessed the street food vendors' knowledge, attitudes, and practices regarding food safety and hygienic practices in the Takoradi Submetro of the Sekondi-Takoradi Metropolitan Assembly, Ghana.

## 2. Materials and Methods

### 2.1. Study Design

This study employed a descriptive cross-sectional design to assess the knowledge, attitude, and practice of food safety and hygiene among street food vendors in the Sekondi-Takoradi Metropolis in August 2022.

### 2.2. Study Site

**Figure 1:**
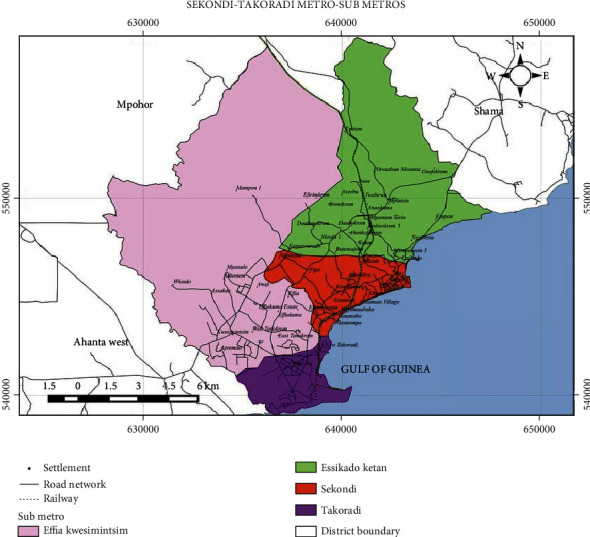
Map of Sekondi-Takoradi Metropolitan Assembly showing the Takoradi Submetro (in pink) (source: Sekondi-Takoradi Metropolitan Assembly (STMA) [13]).

This study was conducted in the Takoradi Submetro of the Sekondi-Takoradi Metropolis in the Western Region of Ghana. The submetro has four communities (Chapel Hill, New Takoradi, Takoradi, and Beach Road) under it and serves as a conduit between the residents of the various towns. Sekondi serves as the administrative centre for the Sekondi-Takoradi Metropolitan Assembly (STMA), which is situated in the south-most portion of the Western Region. Mpohor District, Shama District, Effia-Kwesimintsim Municipal, and the Gulf of Guinea form its northern, eastern, western, and southern boundaries, respectively [13]. With an entire area of 189 km^2^, the STMA is about 130 and 280 kilometers from Accra and La Cote d'Ivoire, the east, respectively. The core area is low lying and is roughly 6 meters above sea level (Figure 1).

### 2.3. Study Population

The study included all street food vendors in the Takoradi Submetro of the STMA who consented to participate in the study. The included street food vendors were people who directly served already cooked food to customers and the owners of the businesses, aged 18 years and above. However, street food vendors who dissented to partake in the study, helpers, and assistants of food venders were excluded from the study.

### 2.4. Sample Size Determination

The sample size was determined using the Cochran (1977) formula, *n* = (*Z*2 *P*(1 − *P*))/*d*2, and was estimated based on the proportion of good food safety, and hygiene practice (*p*) was 62.9% (0.629) from a previous study conducted among Ghanaian street food vendors [1].

Parameters in the formula include the following: *n* which is the sample size, *z* which is the *Z*-score (1.96 at 95% confidence interval), *P* which is the proportion (0.629), and *d* which is the precision or margin of error (0.05).

Hence, sample size is as follows:

(1) $$\begin{array}{c} n=\frac{{\left(1.96\right)}^{2}*0.629*\left(1-0.629\right)}{{\left(0.05\right)}^{2}},\\ n=\frac{3.841*0.629\left(0.371\right)}{0.0025},\\ n=\frac{3.8416*0.2334}{0.0025}, \end{array}$$

where *n* =0.8966/0.0025 and *n* =358.6, approximately 359.

In order to adjust for an anticipated 5% nonresponse rate and also improve statistical power, a total of 408 participants were recruited.

### 2.5. Inclusion and Exclusion Criteria

All food vendors aged 18 years and above who consented to participate were included; however, food vendors below 18 years and those who did not consent to participate were excluded.

### 2.6. Sampling Technique

Recruitment of participants was based on a simple random technique.

### 2.7. Ethical Considerations

Ethical clearance was obtained from The Ghana Institute of Management and Public Administration (GIMPA-REC. [021] 22-23). Prior to data collection, further permission was obtained from the Takoradi Submetropolis of the Sekondi-Takoradi Metropolitan Assembly before the study was started. Written informed consent was obtained from all participants who took part in the study. Likewise, confidentiality and maintenance of anonymity were ensured by using pseudo names.

### 2.8. Data Collection Instrument

A well-structured questionnaire was used to gather sociodemographic and self-reported food safety and hygiene, knowledge, attitude, and practice (KAP) data from study participants. The questionnaire was a close-ended questionnaire. The statements on KAP were adapted from the WHO's Five Keys to Safer Food guidebook for food handlers. The questionnaire was reviewed and validated by professionals. It was pretested among street food vendors who were excluded from the main data collection. The questionnaire was arranged into four sections, sections A, B, C, and D, and each section contained pertinent information.

Section A sought information on respondents' sociodemographic characteristics such as age, religion, sex, and ethnicity. Section B assessed questions on street food vendors' knowledge of food safety and hygiene. It had 12 items (true/false) regarding awareness of food safety, foodborne disease transmission, knowledge of personal hygiene, knowledge of cross-contamination, and knowledge of temperature control. Section C explored questions on the attitude of street food vendors towards food safety and hygiene. It assessed psychological state concerning views, opinion, moral, and characters to act in particular. It contained 10 attitudinal questions with a 3-point Likert scale (2 = agree, 1 = disagree, and 0 = do not know). Section D contained questions on practices of street food vendors towards food safety and hygiene. It had 9 practice items (yes/no).

### 2.9. Data Handling and Statistical Analysis

The data collected were cleaned, coded, and entered into Epi Info version 7 and analyzed using Stata (version 16) software. Categorical variables were expressed as frequency and percentage; Fisher's exact or chi-square and logistic regression analyses were performed to predict the association between study variables and food safety practices. A *p* < 0.05 was considered statistically significant.

## 3. Results

### 3.1. Sociodemographic Characteristics of Food Venders

Table 1 presents the sociodemographic characteristics of the respondents. The mean age of the 406 food vendors was 38.23 (±7.74), with majority (57.1%) of the food vendors aged below 40 years. Majority (90.2%) of the food vendors were females and were married (63.6%). Seventy-nine percent was Christians, with no prior formal training on food vending (63.2%) (Table 1).

### 3.2. Knowledge on Food Safety and Hygiene among Food Vendors

**Figure 2:**
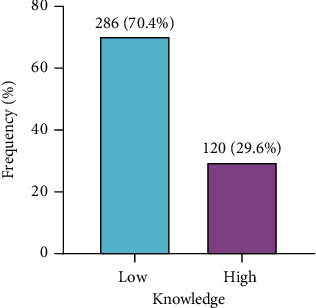
Distribution of knowledge level among study participants.

Table 2 presents knowledge on food safety and hygiene among the street food vendors. The majority (69.7%) reported having heard of food safety and hygiene. Most (86.2%) of them indicated that foodborne illness was caused by food contaminated with microbes. About 96% said that it was safe to wash hands for 1 min, with 99.5% indicating that it was safe to wash ingredient properly. Majority (97.5%) of the food vendors also said that it was hygienic not to touch hair, nose, and mouth while serving food to customers. With an estimated mean score of 7.72 (±1.34) for respondents' knowledge on food safety and hygiene, majority (70.4%) of the respondents had low knowledge on food safety and hygiene (Figure 2).

### 3.3. Attitude towards Food Safety and Hygiene

**Figure 3:**
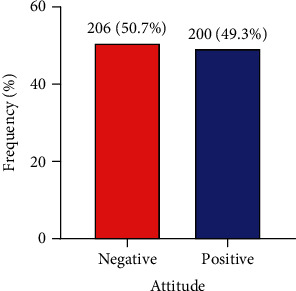
Bar graph depicting the attitude of food vendors towards food safety and hygiene (*N* = 406).

The majority (99.7%) of the food vendors agreed that regular hand cleaning was needful, with 87% of them concurring that cleaning kitchen shelves lessened the danger of infection. Most (93.8%) of the food vendors agreed that they differentiated healthy diets and rotten food. The majority (99%) of the food vendors agreed that it was vital to dispose meals that have gotten to expiring date. The majority (92.4%) of the respondents also agreed that it is important to cough or sneeze inside the elbow if a towel is not available (Table 3). The overall positive attitude was 49.3% (Figure 3).

### 3.4. Food Safety and Hygiene Practices

**Figure 4:**
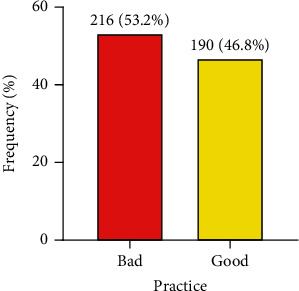
Bar graph depicting food safety and hygiene practice among street food vendors (*N* = 406).

Of the 406 participants, majority (94.8%) indicated that regular cleaning of food and vending site was needful. Most (92.4%) of the food vendors indicated that they washed cooking utensils before preparation of meal, with most (92.1%) of food vendors agreeing that they stored uncooked and cooked meals separately. Also, the majority (85.0%) of vendors reported that they do inspect if a cooked meal was ready by eyeing (Table 4). The overall good food safety and hygiene practices were 46.8% (Figure 4).

### 3.5. Factors Influencing Knowledge on Food Safety and Hygiene

Table 5 presents chi-square test of associations between sociodemographics and knowledge level of food vendors. The study revealed that the associations between sex (*ꭕ*^2^ = 4.52, *p* = 0.034), educational level (*ꭕ*^2^ = 15.88, *p* =0.003), and knowledge level of food vendors were statistically significant.

### 3.6. Factors Influencing Knowledge on Food Venders

Table 6 presents factors influencing knowledge level of food venders on food safety and hygiene. After adjusting for potential confounder in a multivariate binary logistic regression, educational level was the main factor influencing knowledge level of food venders on food safety and hygiene. Food vendors who had attained a senior high school level of education (aOR = 0.37, 95% CI (0.19-0.70), *p* = 0.002) were 63% less likely to have low knowledge of food safety and hygiene as compared to vendors with no formal education. Food vendors who had attained a junior high school level of education (aOR = 0.52, 95% CI (0.27-0.99), *p* = 0.047) were 48% less likely to have low knowledge of food safety and hygiene as compared to vendors with no formal education.

### 3.7. Factors Influencing Attitude of Food Vendors

Table 7 presents factors influencing the attitude of food vendors towards food safety and hygiene. These estimates are presented in two models: crude odds ratio (cOR) and adjusted odds ratio (aOR). Educational level, training on food safety, and knowledge level of food vendors were the main factors influencing the attitude of food vendors towards food safety and hygiene. Food vendors who had attained a senior high school level of education (aOR = 0.37, 95% CI (0.17-0.82), *p* = 0.014) were 63% less likely to have a negative attitude towards food safety and hygiene as compared to vendors with no formal education. Also, food vendors who had training on food safety and hygiene (aOR = 0.50, 95% CI (0.29-0.84), *p* = 0.010) were 50% less likely to have a negative attitude of food vendors towards food safety and hygiene as compared to vendors who had no training. Food vendors with a high level of knowledge (aOR = 0.33, 95% CI (0.20-0.54), *p* = 0.001) were 67% less likely to have a negative attitude towards food safety and hygiene as compared to vendors with a low level of knowledge.

### 3.8. Factors Influencing Practice of Food Safety and Hygiene among Food Vendors

Table 8 presents factors influencing the practice of food safety and hygiene among food vendors. These estimates are presented in two models: crude odds ratio (cOR) and adjusted odds ratio (aOR). Educational level, family size, knowledge level of food vendors, and their attitudes were the main factors influencing the practice of food safety and hygiene among food vendors. Food vendors who had attained junior high school level of education (aOR = 6.20, 95% CI (2.78-13.87), *p* = 0.001) were 6 times more likely to have good practice of food safety and hygiene as compared with those with no formal education. Also, food vendors with a family size of 6-7 (aOR = 4.37, 95% CI (2.02-9.43), *p* = 0.001) were 4 times more likely to have good practices of food safety and hygiene as compared with those with a family size of 1-3. Food vendors with a high level of knowledge (aOR = 4.70, 95% CI (2.77-7.98), *p* = 0.001) were 4 times more likely to practice good food safety and hygiene measures as compared to those with a low level of knowledge. Food vendors who had a positive attitude towards food safety and hygiene (aOR = 1.76, 95% CI (1.08-2.87), *p* = 0.023) were 2 times more likely to have good practices of food safety and hygiene as compared with those with a negative attitude.

## 4. Discussion

We examined the knowledge, attitudes, and practice regarding food safety and hygiene among street food vendors in the Sekondi-Takoradi Metropolis of Ghana. Our findings showed that the level of knowledge was low among 70.4% of the food vendors and 51% had negative attitudes towards food safety and hygiene. Food hygiene practices were also poor among 52.3% of the participants. Level of education and sex significantly influenced knowledge on food safety and hygiene. Level of education, receipt of training on food safety and hygiene, and level of knowledge were the factors influencing attitude towards food safety and hygiene. Moreover, level of education, family size, level of knowledge, and attitude towards food safety and hygiene were the predictors of food hygiene practices among the street food vendors.

Comparatively, a study in Egypt among 994 food vendors found that only 39.2% of the participants had good food safety knowledge [14]. A similar study in Malaysia on the assessment of the knowledge, attitudes, and practices in food safety among food handlers engaged in food courts found that 58.3% of them had poor knowledge [15]. On the contrary, a recent study in Ghana by Tuglo et al. [1] reported that 67.3% of the food vendors had good knowledge [1], which is higher compared to the proportion of knowledge observed in the present study. This indicates that knowledge regarding food safety has reduced drastically and calls for immediate action. Another study by Rahman et al. [16] among street vendors in Kuching City revealed that the respondents had good knowledge (41.6%), positive attitude (19.1%), and good practice (10.8%) [16] which is inconsistent with our findings. The differences observed between the studies could be due to variations in sample size and geographical distribution.

The present study found education as the independent predictor of knowledge among food vendors, which contradicts earlier findings by Odonkor et al. [17], which stated that those who had attained primary education had better food safety and hygiene practices compared to the other levels of education [17]. Other studies have shown that the odds of having good knowledge were lower among food vendors who had not attained any formal education or had had a primary education compared to those with higher educational status [18]. This highlights the fact that food vendors should be encouraged to attain at least a basic education. A study in Indonesia by Cempaka et al. reported a significant association between level of education and the participation in food hygiene training on the KAP level [19]. That same study demonstrated that food handlers with excellent knowledge levels were 3 times more likely to have excellent food hygiene practices [19]. Moreover, a study in Egypt among 994 food handlers found that higher education was a strong predictor of good food safety knowledge [20], which concurs with our findings. Education increases one's chance to get better information regarding food safety in comparison to those who are noneducated. Also, individuals who are educated can read texts on food safety from leaflets, posters, etc., which may improve their knowledge of food safety. A low level of education reduces awareness; however, the higher one gets educated the better the knowledge, which affects their attitude and eventually may reflect into good hygiene practices.

Our study also revealed that vendors who had training on food safety and hygiene were significantly associated with lower chances of having negative attitude towards food safety and hygiene. Similarly, a Malaysian study reported that prior training and knowledge were significantly associated with attitude towards food safety and hygiene practices [16]. Furthermore, similar studies have reported a significant association between training, knowledge, attitude, and food safety and hygiene practice [15, 21].

This association reveals that food handlers with good knowledge have good attitudes and good practices. Thus, actions geared towards the improvement in the knowledge of food vendors will enhance their food safety and hygienic practices. Collaborations of municipal assemblies with other agencies are strongly encouraged to strengthen, sustain, and organize regular training programs for new entrants and existing food vendors as well as retraining of trainers to equip them adequately with knowledge and skills to enable them to effectively facilitate training programs for food vendors. One major limitation of this study was its relatively small sample size; however, it was good enough to draw meaningful conclusions.

## 5. Conclusions

Knowledge, attitude, and practices regarding food safety and hygiene were poor among street food vendors at the Sekondi-Takoradi Metropolitan area of Ghana. Our findings suggest that future initiatives should focus on establishing training programs for food vendors within the metropolitan assembly to improve their knowledge on food safety and hygienic practices.

## Figures and Tables

**Table 1:** Sociodemographic characteristics of respondents.

Variable	Frequency (*N* = 406)	Percentage (%)
Age		
Estimated mean (SD)	38.23 (±7.74)	
<40	232	57.1
40+	174	42.9
Sex		
Female	366	90.2
Male	40	9.8
Educational level		
No formal education	85	20.9
Primary	47	11.6
Junior high school	118	29.1
Senior high school	101	24.9
Tertiary	55	13.5
Marital status		
Never married	97	23.9
Married	258	63.6
Separated/divorced	22	5.4
Widowed	29	7.1
Religion		
Christian	321	79.0
Muslim	79	19.5
Traditionalist	6	1.5
Ethnicity		
Akan	223	54.9
Ewe	59	14.5
Ga/Dangme	36	8.9
Fante	25	6.2
Mole-Dagbani	63	15.5
Family size		
1-3	129	31.8
4-5	159	39.2
6-7	84	20.7
8+	34	8.3
Duration of food vending		
Less than 2 months	32	7.9
2-4 months	116	28.6
4-6 months	128	31.5
More than 6 months	130	32.0
Formal training on food vending		
No	253	62.3
Yes	153	37.7

Data presented as frequency and percentages.

**Table 2:** Knowledge of food safety and hygiene among street food vendors.

Variable	Frequency (*N* = 406)	Percentage (%)
Have you ever heard of food safety and hygiene?		
No	123	30.3
Yes	283	69.7
Foodborne illnesses are caused by food contaminated with microbes		
False	9	2.2
True	350	86.2
Do not know	47	11.6
Clean and healthy food handlers carry harmful microbes on their bodies		
False	140	34.5
True	155	38.2
Do not know	111	27.3
No need to tie long hair when handling unpackaged food		
False	239	58.9
True	117	28.8
Do not know	50	12.3
It is safe to wash hands for 1 min before touching food		
False	9	2.2
True	391	96.3
Do not know	6	1.5
It is safe to wash ingredient properly before cooking		
False	1	0.2
True	404	99.6
Do not know	1	0.2
All microbes are harmful		
False	65	16.0
True	233	57.4
Do not know	108	26.6
Food should be sold in clean environment		
True	405	99.8
Do not know	1	0.2
It is good to use different chopping boards for uncooked and prepared food		
False	52	12.8
True	293	72.2
Do not know	61	15.0
Hygienic not to touch hair, nose, and mouth while serving food to customers		
False	7	1.7
True	396	97.6
Do not know	3	0.7
Safe to keep hot food hot and cold food cold		
False	13	3.2
True	389	95.8
Do not know	4	1.0
Excess food should be kept at required temperature		
False	13	3.2
True	393	96.8

Data presented as frequency and percentages (%).

**Table 3:** Attitude towards food safety and hygiene among food vendors.

Variable	Frequency (*N* = 406)	Percentage (%)
Regular hand cleaning is needful		
Disagree	1	0.2
Agree	405	99.8
Cleaning kitchen shelves lessen the danger of infection		
Disagree	53	13.1
Agree	353	86.9
Separating uncooked and prepared meal stops infection		
Disagree	77	19.0
Agree	329	81.0
Differentiate healthy diets and rotten food by eyeing		
Disagree	25	6.2
Agree	381	93.8
Using different knives enhances food safety		
Disagree	92	22.7
Agree	314	77.3
It is appropriate to cough or sneeze inside the elbow if towel is not available		
Disagree	31	7.6
Agree	375	92.4
Checking meal for cleanliness and healthiness is important		
Disagree	2	0.5
Agree	404	99.5
Vital to dispose of meals that have gotten to expiring date		
Disagree	4	1.0
Agree	402	99.0
It is unhealthy to leave prepared meal out of the fridge over 2 hours		
Disagree	124	30.5
Agree	282	69.5

Data presented as frequency and percentages (%).

**Table 4:** Food safety and hygiene practices among food vendors (*N* = 406).

Variable	Frequency (*n* = 406)	Percentage (%)
Regular cleaning is needful		
No	21	5.2
Yes	385	94.8
Wash cooking utensils before preparation of meal		
No	31	7.6
Yes	375	92.4
Store uncooked and cooked meals separately		
No	32	7.9
Yes	374	92.1
Keep prepared food at room temperature for not more than 2 hours		
No	176	43.3
Yes	230	56.7
Check and dispose expired food products		
No	9	2.2
Yes	397	97.8
Do you inspect if a cooked meal is ready by eyeing?		
No	61	15.0
Yes	345	85.0
Do you examine if a grilled meal is ready by touching?		
No	93	22.9
Yes	313	77.1
Use similar kitchen cloth to clean shelves and hands		
No	230	56.6
Yes	176	43.4

Data presented as frequency and percentages (%).

**Table 5:** Bivariable analysis of factors influencing knowledge on food safety and hygiene.

Explanatory variable	Level of knowledge (*N* = 406)		*ꭕ* ^2^ (*p* value)
	Low (*n* = 286)	High (*n* = 120)	
Age (in completed years)			
<40	161 (69.4)	71 (30.6)	
40+	125 (71.8)	49 (28.2)	0.29 (0.594)
Sex			
Female	252 (68.9)	114 (31.1)	
Male	34 (85.0)	6 (15.0)	4.52 **(0.034)**
Educational level			
No formal education	67 (78.8)	18 (21.2)	
Primary	34 (72.3)	13 (27.7)	
Junior high school	78 (66.1)	40 (33.9)	
Senior high school	60 (59.4)	41 (40.6)	
Tertiary	47 (85.5)	8 (14.5)	15.88 **(0.003)**
Marital status			
Single	66 (68.0)	31 (32.0)	
Married	182 (70.5)	76 (29.5)	
Separated/divorced	14 (63.6)	8 (36.4)	
Widow	24 (82.8)	5 (17.2)	2.87 (0.412)
Religion			
Christianity	225 (70.1)	96 (29.9)	
Islam	55 (69.6)	24 (30.4)	
Traditionalist	6 (100)	0	2.56 (0.353)
Family size			
1-3	94 (72.9)	35 (27.1)	
4-5	107 (67.3)	52 (32.7)	
6-7	60 (71.4)	24 (28.6)	
8 or more	25 (73.5)	9 (26.5)	1.32 (0.725)
Duration of food vending			
3-4 months	86 (74.1)	30 (25.9)	
5-6 months	87 (68.0)	41 (32.0)	
Less than 2 months	21 (65.6)	11 (34.4)	
More than 6 months	92 (70.8)	38 (29.2)	1.50 (0.682)
Training on food safety			
No	185 (73.1)	68 (26.9)	
Yes	101 (66.0)	52 (36.0)	2.31 (0.128)

Chi-square *p* values are presented, bolded *p* values were statistically significant, and *p* < 0.05 was considered statistically significant.

**Table 6:** Multivariable analysis of factors affecting the knowledge level of food vendors.

Variable	Low knowledge (*n* = 286)	cOR (95% CI)	*p* value	aOR (95% CI)	*p* value
Sex					
Female (Ref)	252 (88.1)	1.00	—	1.00	—
Male	34 (11.9)	2.56 (1.05-6.28)	0.039	2.41 (0.94-6.20)	0.069
Educational level					
No formal education (Ref)	67 (23.4)	1.00	—	1.00	—
Primary	34 (11.9)	0.70 (0.31-1.60)	0.401	0.70 (0.31-1.61)	0.403
Junior high school	78 (27.3)	0.52 (0.28-0.99)	0.049	0.52 (0.27-0.99)	**0.047**
Senior high school	60 (21.0)	0.39 (0.20-0.76)	0.005	0.36 (0.19-0.70)	**0.002**
Tertiary	47 (16.4)	1.58 (0.63-3.93)	0.327	1.26 (0.49-3.21)	0.634

Ref: reference; cOR: crude odds ratio; aOR: adjusted odds ratio. *p* < 0.05 was considered statistically significant, and bolded *p* values are significant.

**Table 7:** Multivariable analysis of factors affecting the attitude of food vendors.

Variable	Negative attitude (*N* = 206)	cOR (95% CI)	*p* value	aOR (95% CI)	*p* value
Age (years)					
<40 (Ref)	99 (48.1)	1.00	—	1.00	—
>40	107 (51.9)	2.15 (1.44-3.20)	<0.001	1.52 (0.90-2.56)	0.118
Educational level					
No formal education (Ref)	60 (29.1)	1.00	—	1.00	—
Primary	35 (17.0)	1.22 (0.54-2.72)	0.635	1.99 (0.82-4.84)	0.130
Junior high school	56 (27.2)	0.38 (0.21-0.68)	0.001	0.65 (0.32-1.30)	0.220
Senior high school	30 (14.6)	0.18 (0.09-0.33)	<0.001	0.37 (0.17-0.82)	**0.014**
Tertiary	25 (12.1)	0.35 (0.17-0.70)	0.003	0.75 (0.29-1.96)	0.554
Marital status					
Single (Ref)	44 (21.4)	1.00	—	1.00	—
Married	127 (61.6)	1.17 (0.73-1.87)	0.516	0.72 (0.41-1.25)	0.238
Separated/divorced	12 (5.8)	1.45 (0.57-3.66)	0.437	0.66 (0.23-1.94)	0.499
Widow	23 (11.2)	4.62 (1.73-12.34)	0.002	1.45 (0.46-4.58)	0.523
Training on food safety					
No (Ref)	152 (73.8)	1.00	—	1.00	—
Yes	54 (26.2)	0.36 (0.24-9.55)	<0.001	0.50 (0.29-0.84)	**0.010**
Knowledge level					
Low (Ref)	169 (82.0)	1.00	—	1.00	—
High	37 (18.0)	0.31 (0.19-0.49)	<0.001	0.33 (0.20-0.54)	**<0.001**

Ref: reference; cOR: crude odds ratio; aOR: adjusted odds ratio. *p* < 0.05 was considered statistically significant, and bolded *p* values are significant.

**Table 8:** Multivariable analysis of factors affecting food safety and hygiene practices among food vendors.

Variable	Practice (*n* = 406)		*ꭕ* ^2^ (*p* value)/Fisher's exact	cOR (95% CI) *p* value	aOR (95% CI) *p* value
	Bad (*n* = 216)	Good (*n* = 190)			
Sex					
Female	186 (50.8)	180 (49.2)		Reference	Reference
Male	30 (75.0)	10 (25.0)	8.47 (0.004)	0.34 (0.16-0.73) 0.005	0.54 (0.24-1.29) 0.156
Educational level					
No formal education	61 (71.8)	24 (28.2)		Reference	Reference
Primary	23 (48.9)	24 (51.1)		2.65 (1.26-5.57) 0.010	4.99 (2.02-12.34) **0.001**
Junior high school	52 (44.1)	66 (55.9)		3.23 (1.78-5.85) <0.001	5.83 (2.70-12.57) **<0.001**
Senior high school	41 (40.6)	60 (59.4)		3.72 (2.01-6.89) <0.001	6.20 (2.78-13.87) **<0.001**
Tertiary	39 (70.9)	16 (29.1)	29.44 (<0.001)	1.04 (0.49-2.21) 0.913	3.21 (1.21-8.53) **0.019**
Family size					
1-3	85 (65.9)	44 (34.1)		Reference	Reference
4-5	82 (51.6)	77 (48.4)		1.81 (1.12-2.93) 0.015	1.88 (1.02-3.44) **0.042**
6-7	33 (39.3)	51 (60.7)		2.99 (1.68-5.28) <0.001	4.37 (2.02-9.43) **<0.001**
8 or more	16 (47.1)	18 (52.9)	15.56 (0.001)	2.17 (1.01-4.67) 0.047)	4.41 (1.43-13.53) **0.010**
Duration of food vending					
3-4 months	75 (64.7)	41 (35.3)		Reference	Reference
5-6 months	65 (50.8)	63 (49.2)		1.77 (1.06-2.97) 0.029	1.46 (0.78-2.71) 0.235
Less than 2 months	15 (46.9)	17 (53.1)		2.07 (0.94-4.58) 0.071	2.18 (0.88-5.40) 0.091
More than 6 months	61 (46.9)	69 (53.1)	8.99 (0.029)	2.07 (1.24-3.46) 0.006	2.04 (1.00-4.17) 0.051
Knowledge level					
Low	186 (65.0)	100 (35.0)		Reference	Reference
High	30 (25.0)	90 (75.0)	54.42 (<0.001)	5.58 (3.46-9.01) <0.001	4.70 (2.77-7.98) **<0.001**
Attitude					
Negative	132 (64.1)	74 (35.9)		Reference	Reference
Positive	84 (42.0)	116 (58.0)	19.87 (<0.001)	2.46 (1.65-3.67) <0.001	1.76 (1.08-2.87) **0.023**

cOR: crude odds ratio; aOR: adjusted odds ratio. *p* < 0.05 was considered statistically significant, and bolded *p* values are significant.

## Data Availability

All data generated or analyzed during this study are included in this article and can be requested from the corresponding author.
